# Supplementary figures and images for: T-cell receptor (TCR) signaling promotes the assembly of RanBP2/RanGAP1-SUMO1/Ubc9 nuclear pore subcomplex via PKC-θ-mediated phosphorylation of RanGAP1
Source: eLife. 2021 Jun 10;10:e67123. doi: 10.7554/eLife.67123 (PMC8225385; doi:10.7554/eLife.67123)

**Figure 1**

**Figure 1A**

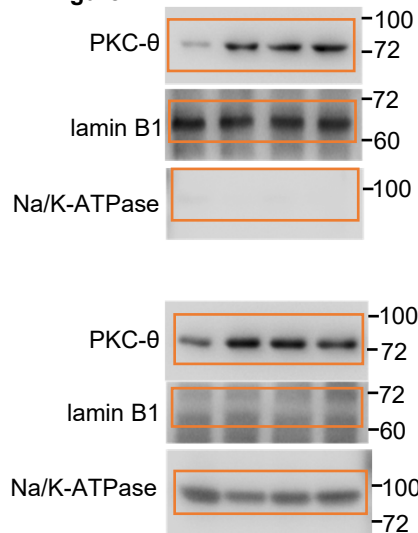

**Figure 1B**

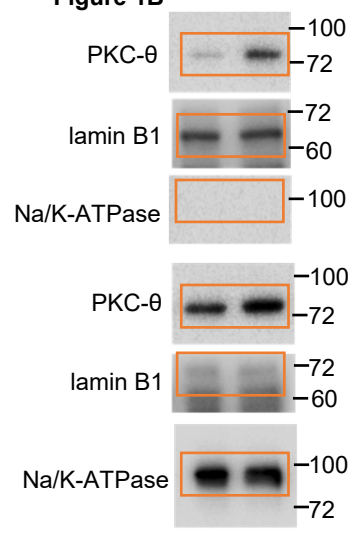

**Figure 1E**

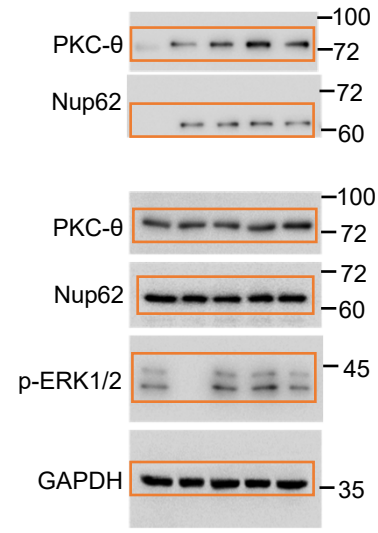

**Figure 1F**

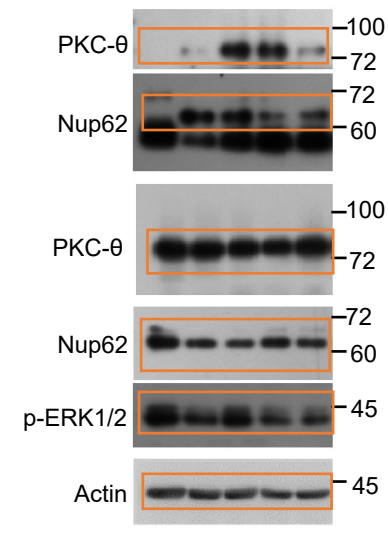

**Figure 1H**

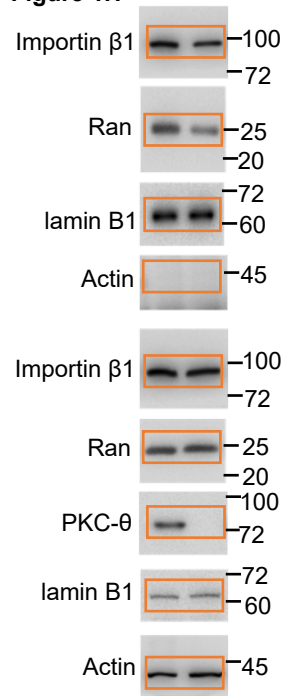

**Figure 1I**

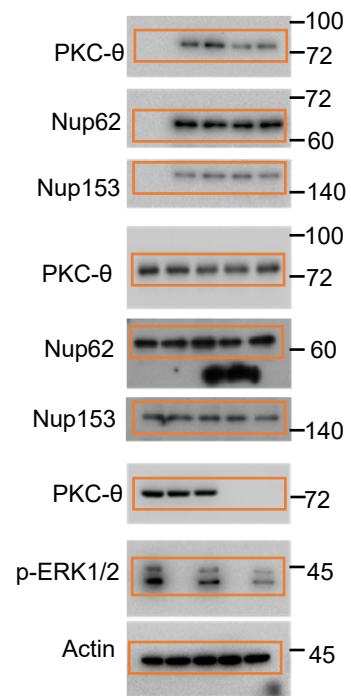

**Figure 1K**

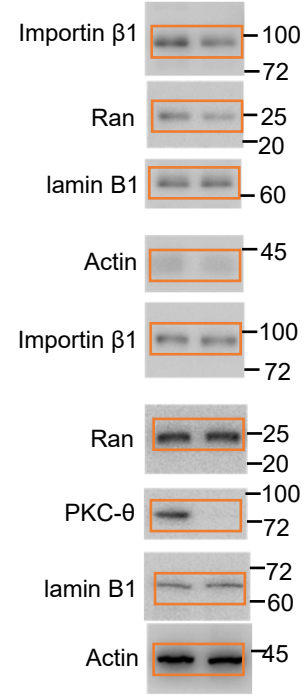

**Figure 1L**

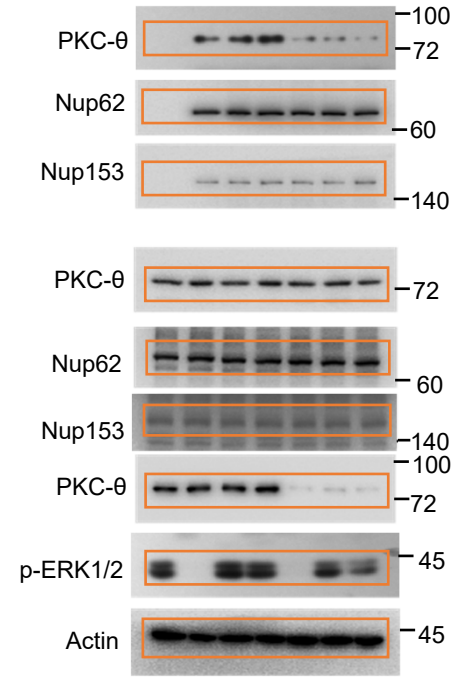

Supplement: Figure 1—source data 1. [file elife-67123-fig1-data1.pdf]

## Figure 1-figure supplement 1

**Figure S1M**

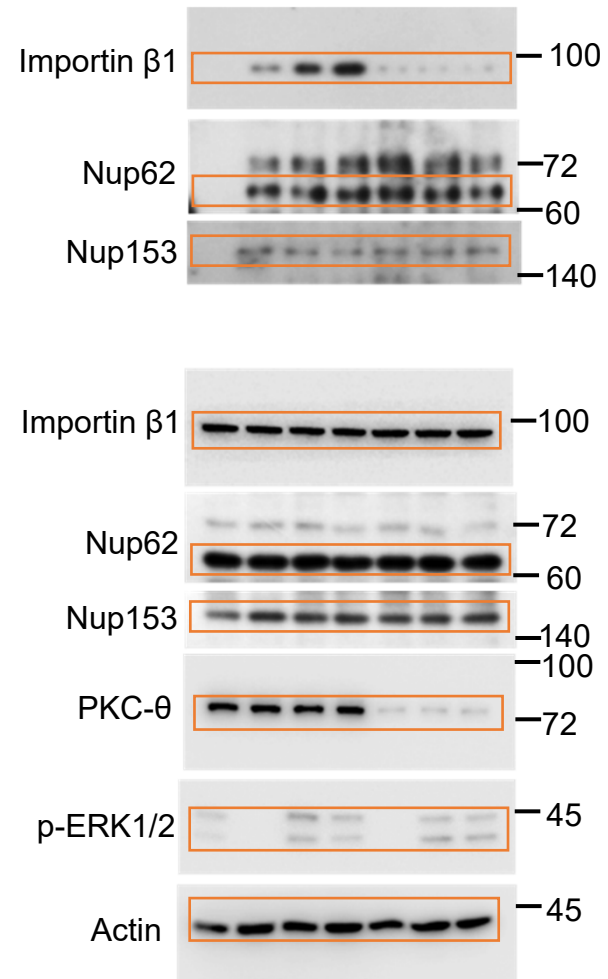

Supplement: Figure 1—figure supplement 1—source data 1. [file elife-67123-fig1-figsupp1-data1.pdf]

**Figure 2**

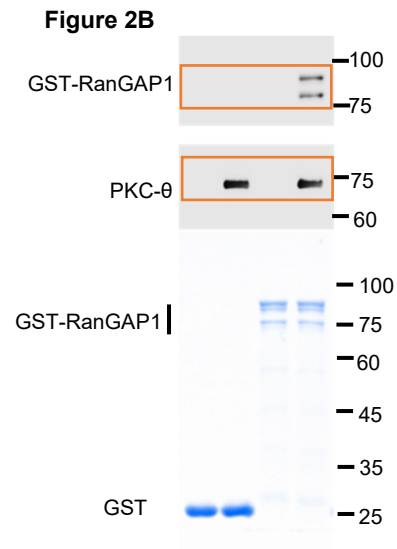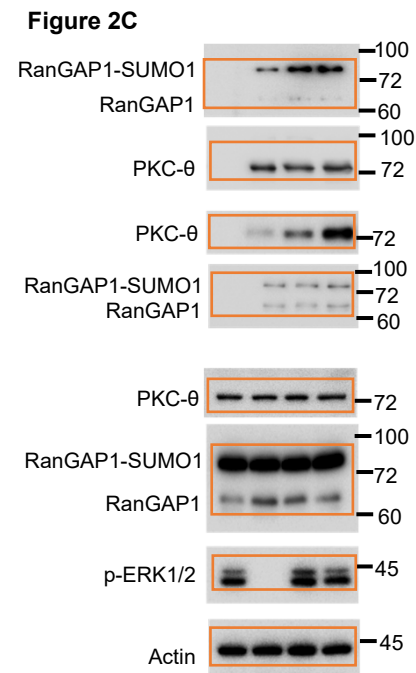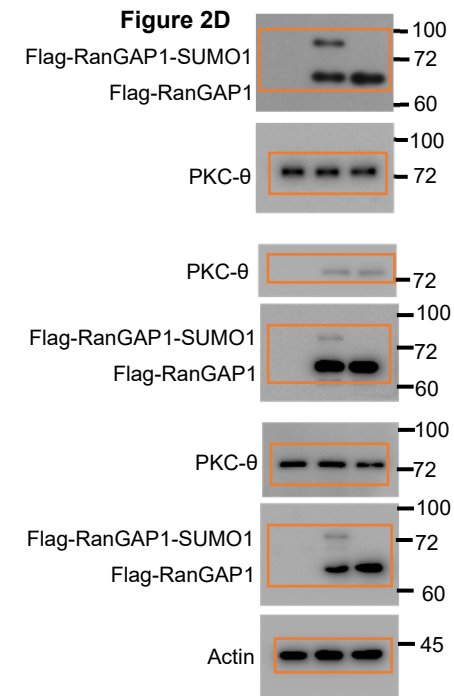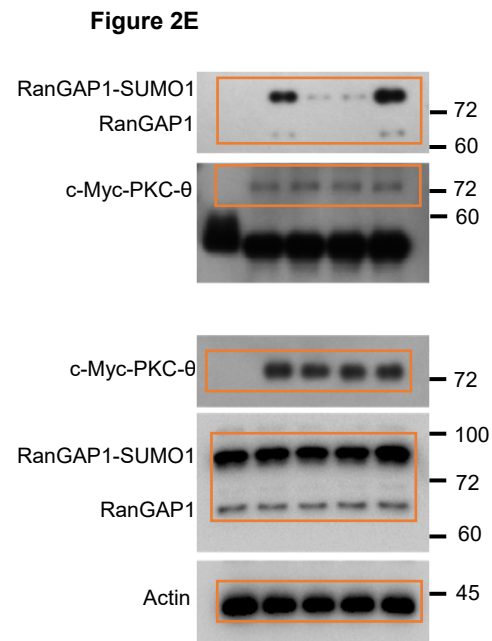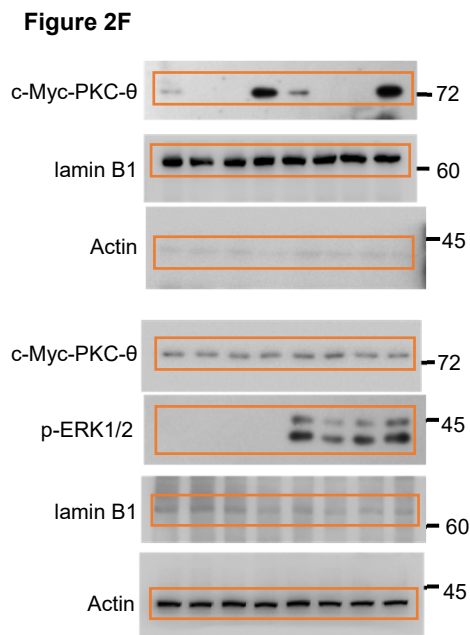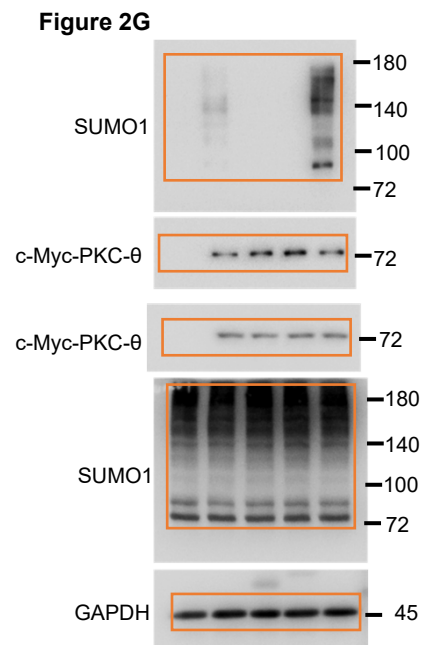

Supplement: Figure 2—source data 1. [file elife-67123-fig2-data1.pdf]

## Figure 2-figure supplement 1

Figure S2A

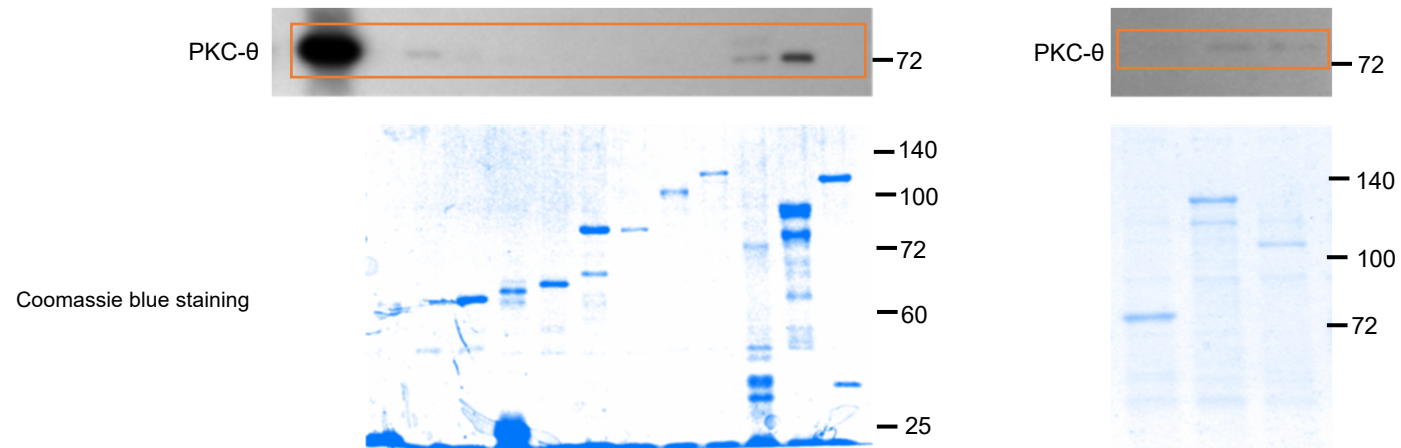

Figure S2D

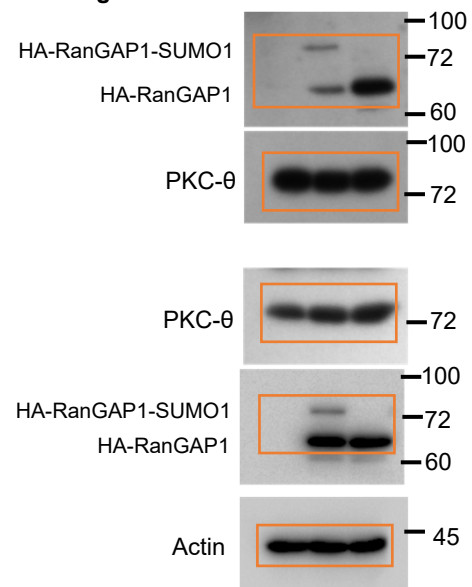

Figure S2F

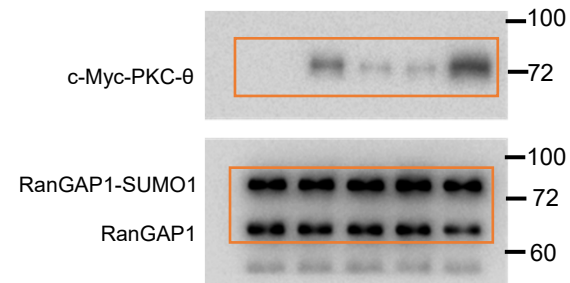

Supplement: Figure 2—figure supplement 1—source data 1. [file elife-67123-fig2-figsupp1-data1.pdf]

**Figure 3**

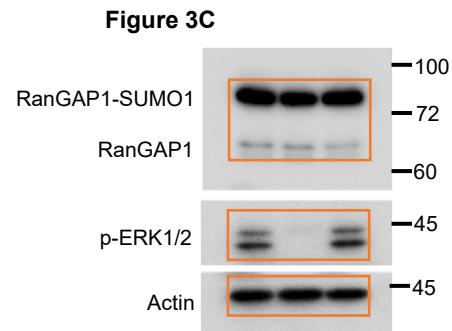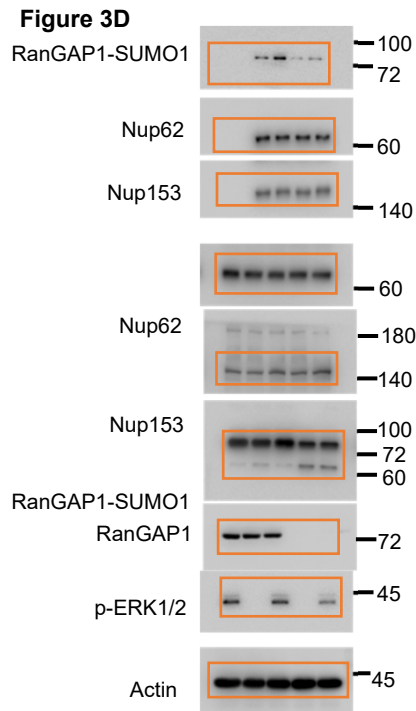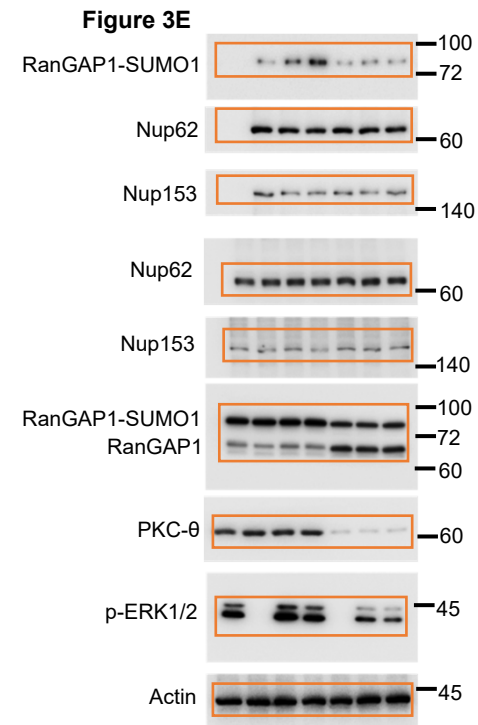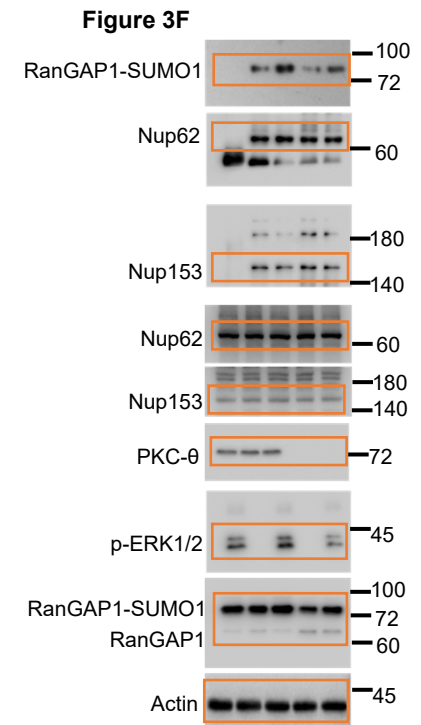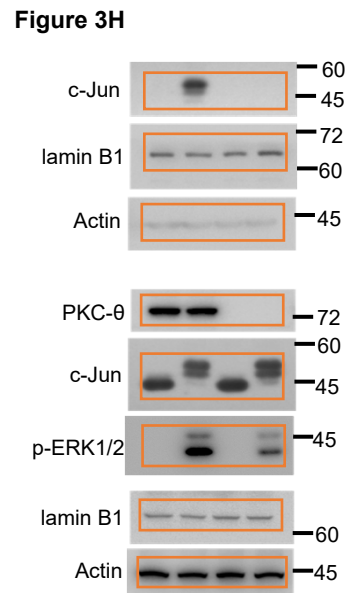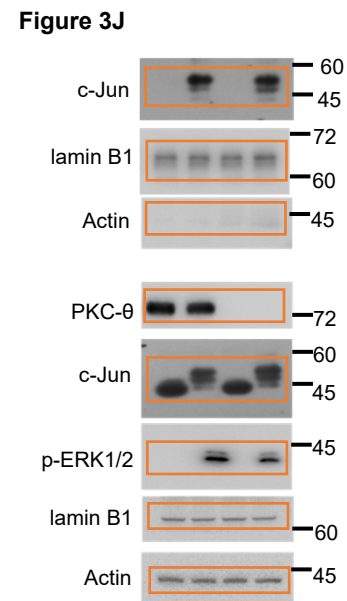

Supplement: Figure 3—source data 1. [file elife-67123-fig3-data1.pdf]

## Figure 3-figure supplement 1

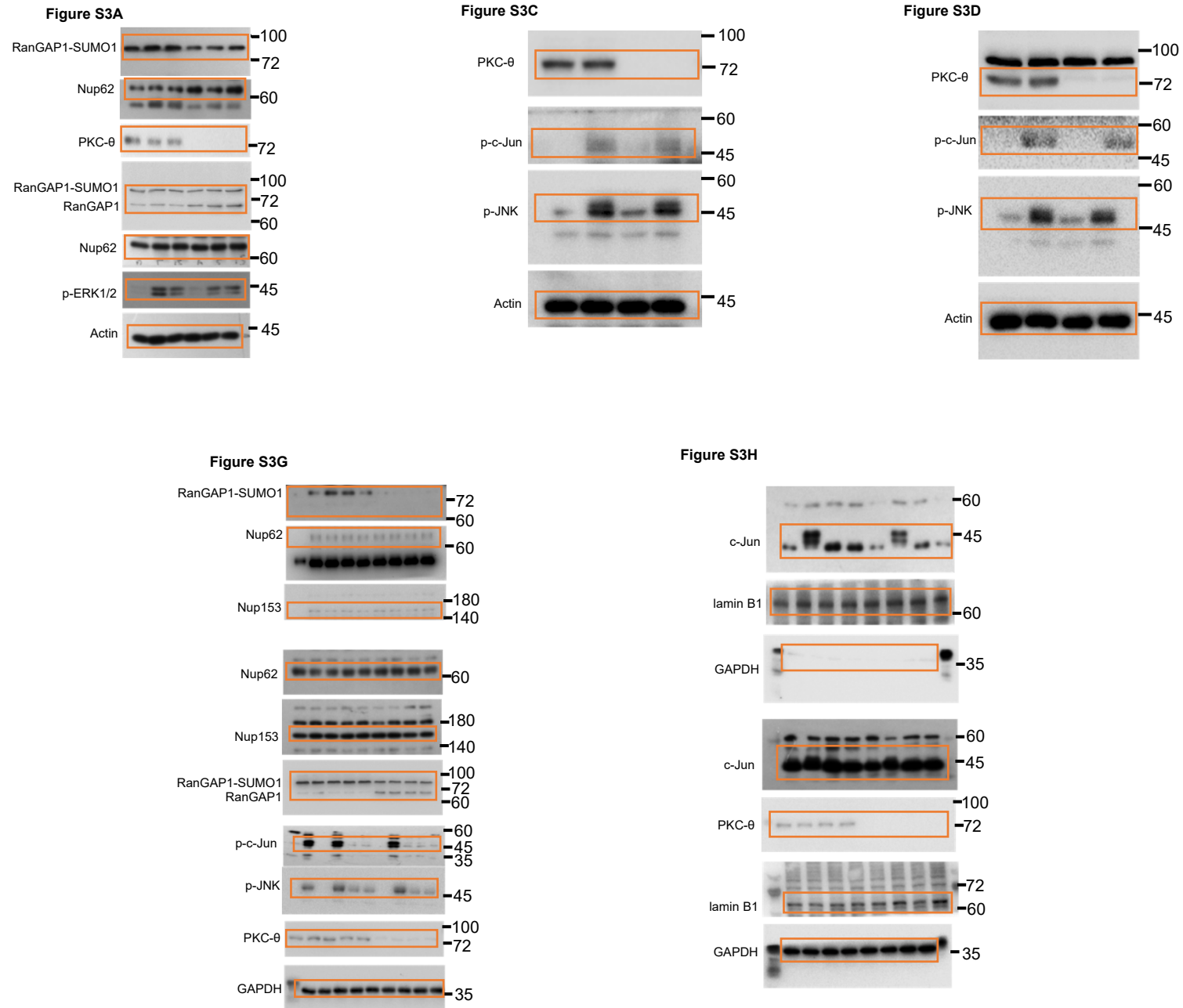

Supplement: Figure 3—figure supplement 1—source data 1. [file elife-67123-fig3-figsupp1-data1.pdf]

Figure 4

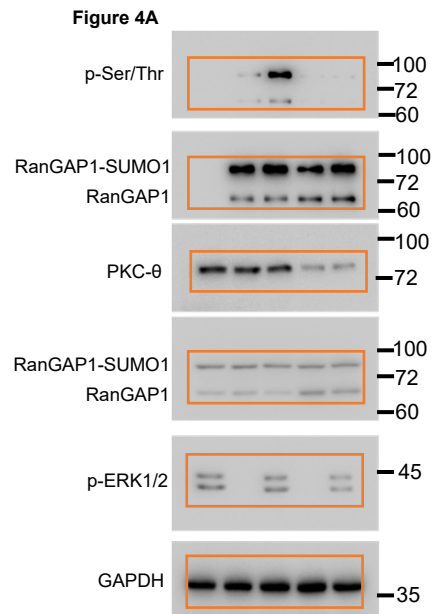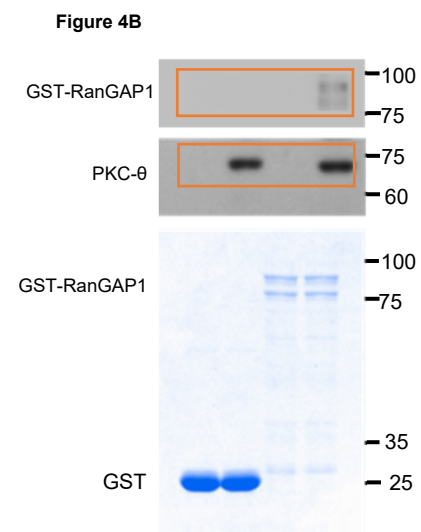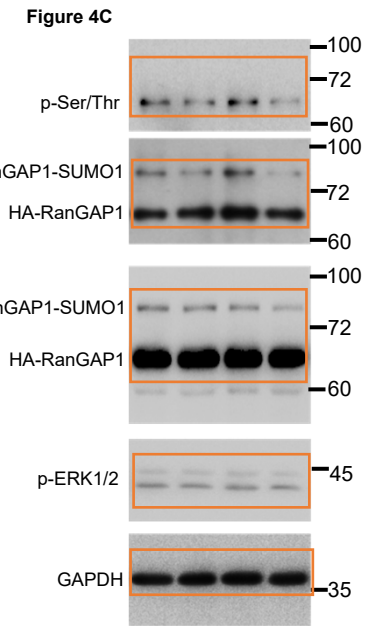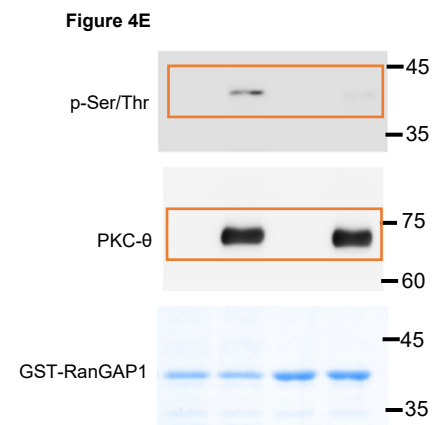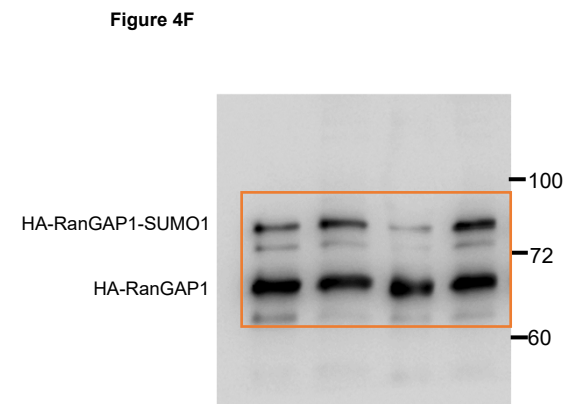

Supplement: Figure 4—source data 1. [file elife-67123-fig4-data1.pdf]

## Figure 4-figure supplement 1

Figure S4A

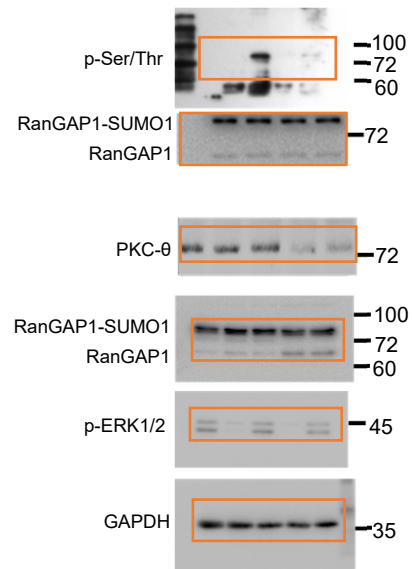

Figure S4C

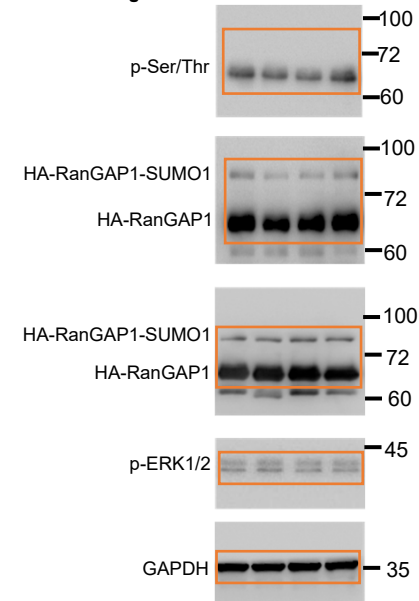

Figure S4E

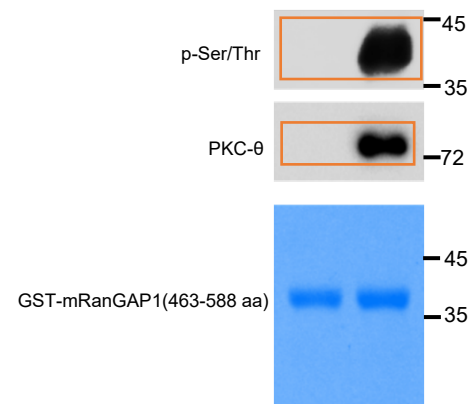

Figure S4F

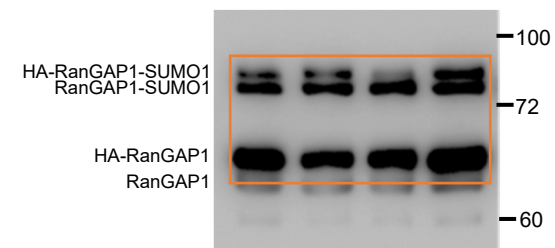

Supplement: Figure 4—figure supplement 1—source data 1. [file elife-67123-fig4-figsupp1-data1.pdf]

**Figure 5**

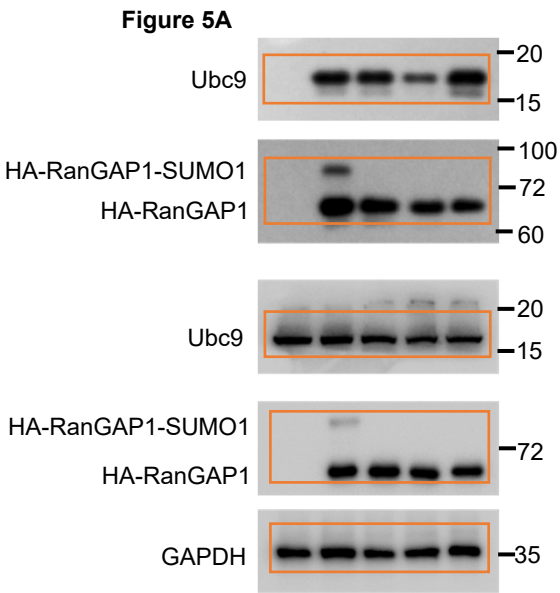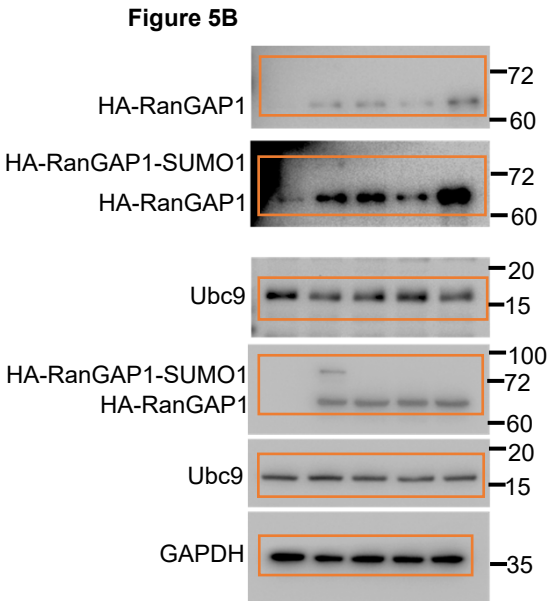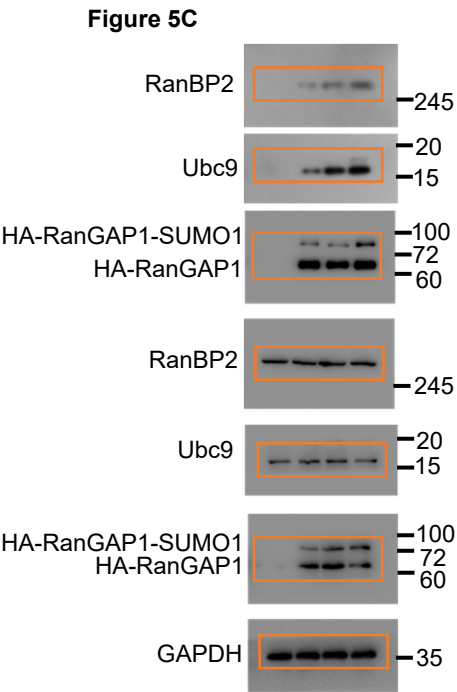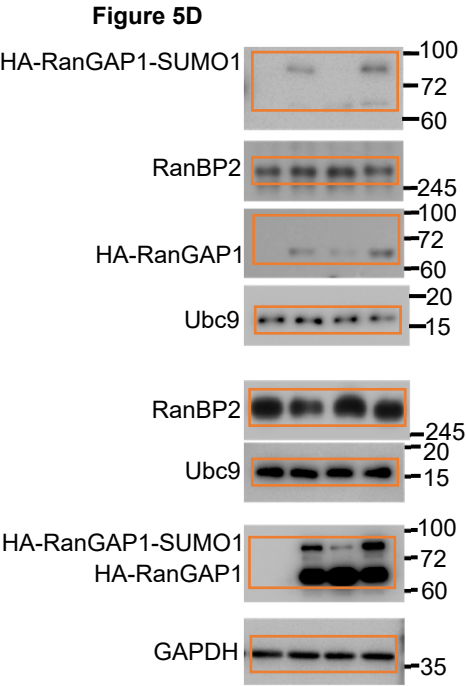

Supplement: Figure 5—source data 1. [file elife-67123-fig5-data1.pdf]

Figure 5-figure supplement 1

Figure S5A

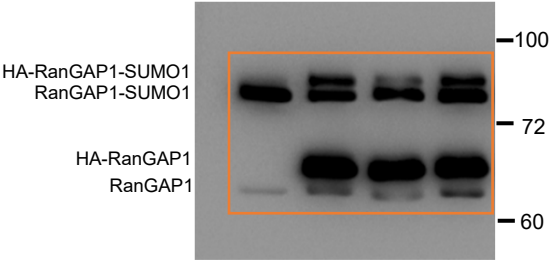

Supplement: Figure 5—figure supplement 1—source data 1. [file elife-67123-fig5-figsupp1-data1.pdf]

**Figure 6A**

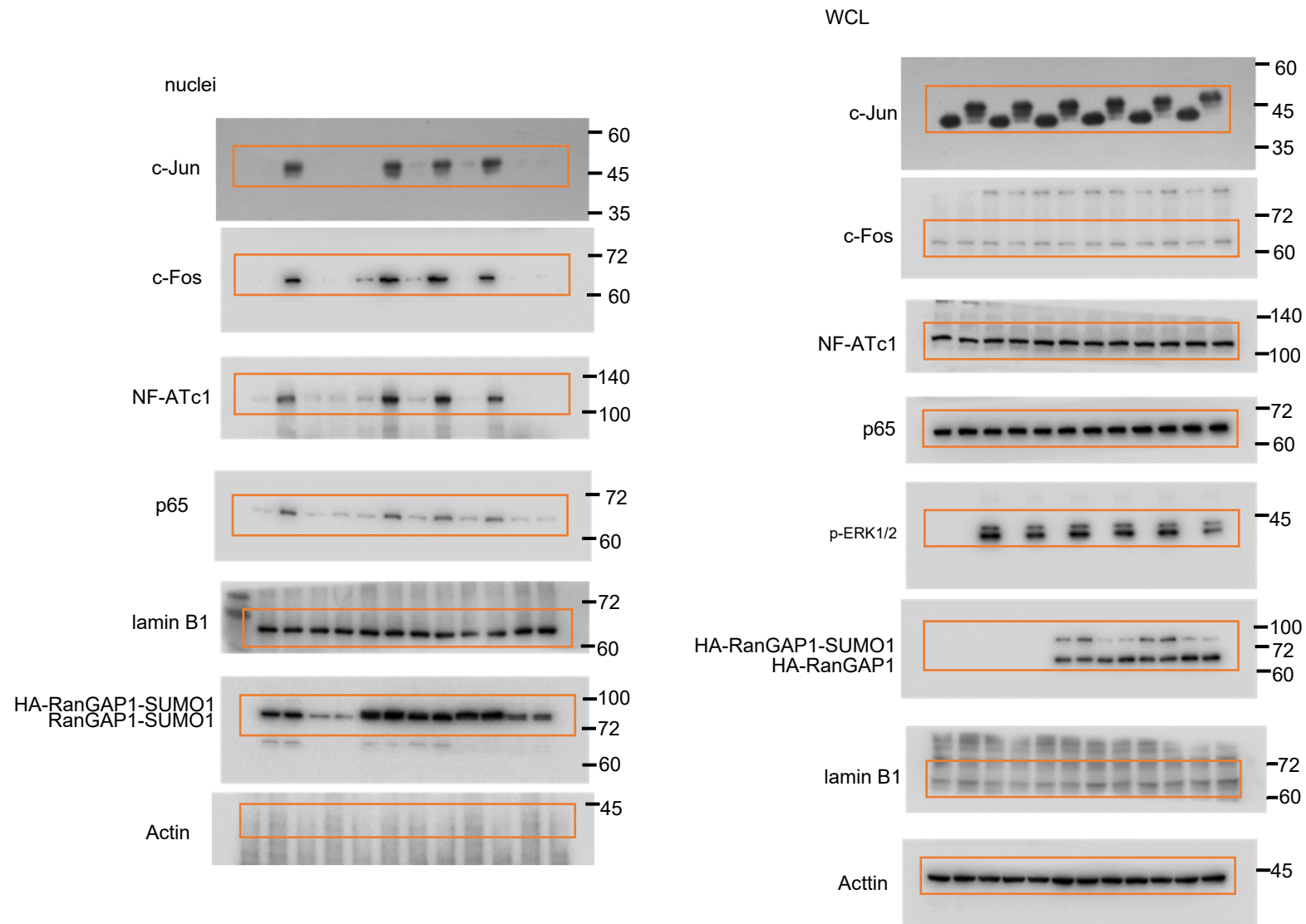

Supplement: Figure 6—source data 1. [file elife-67123-fig6-data1.pdf]

## Figure 6-figure supplement 1

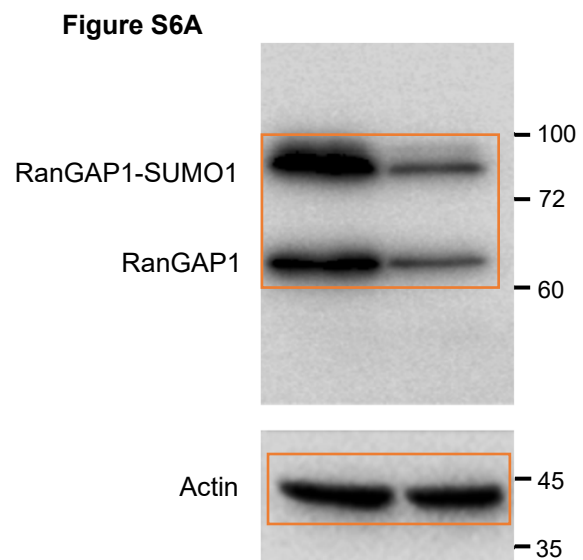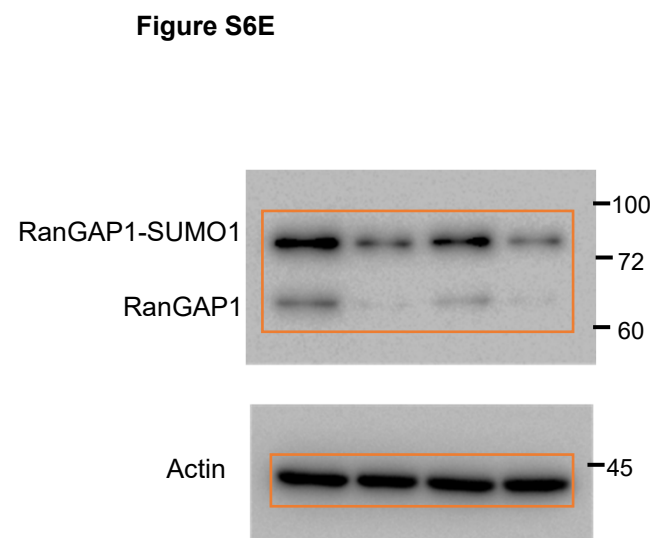

Supplement: Figure 6—figure supplement 1—source data 1. [file elife-67123-fig6-figsupp1-data1.pdf]

**Figure 7A**

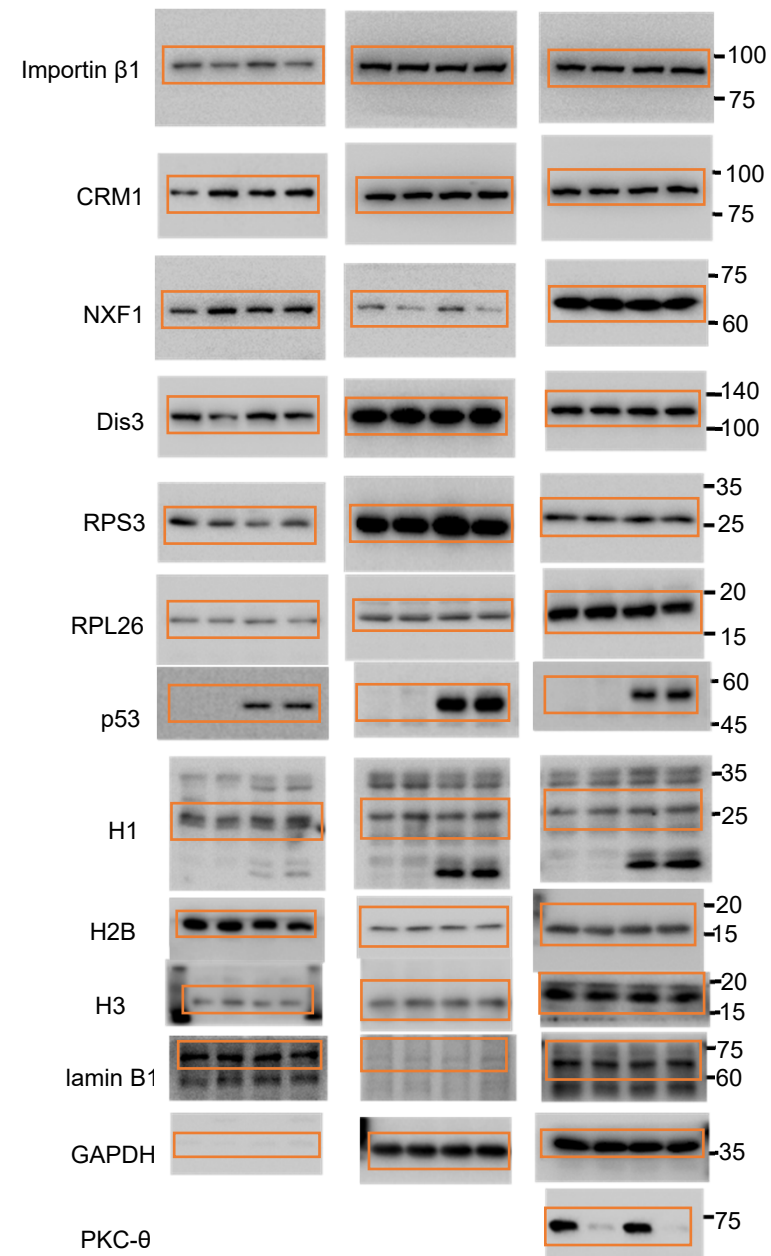

Supplement: Figure 7—source data 1. [file elife-67123-fig7-data1.pdf]

**Figure 8A**

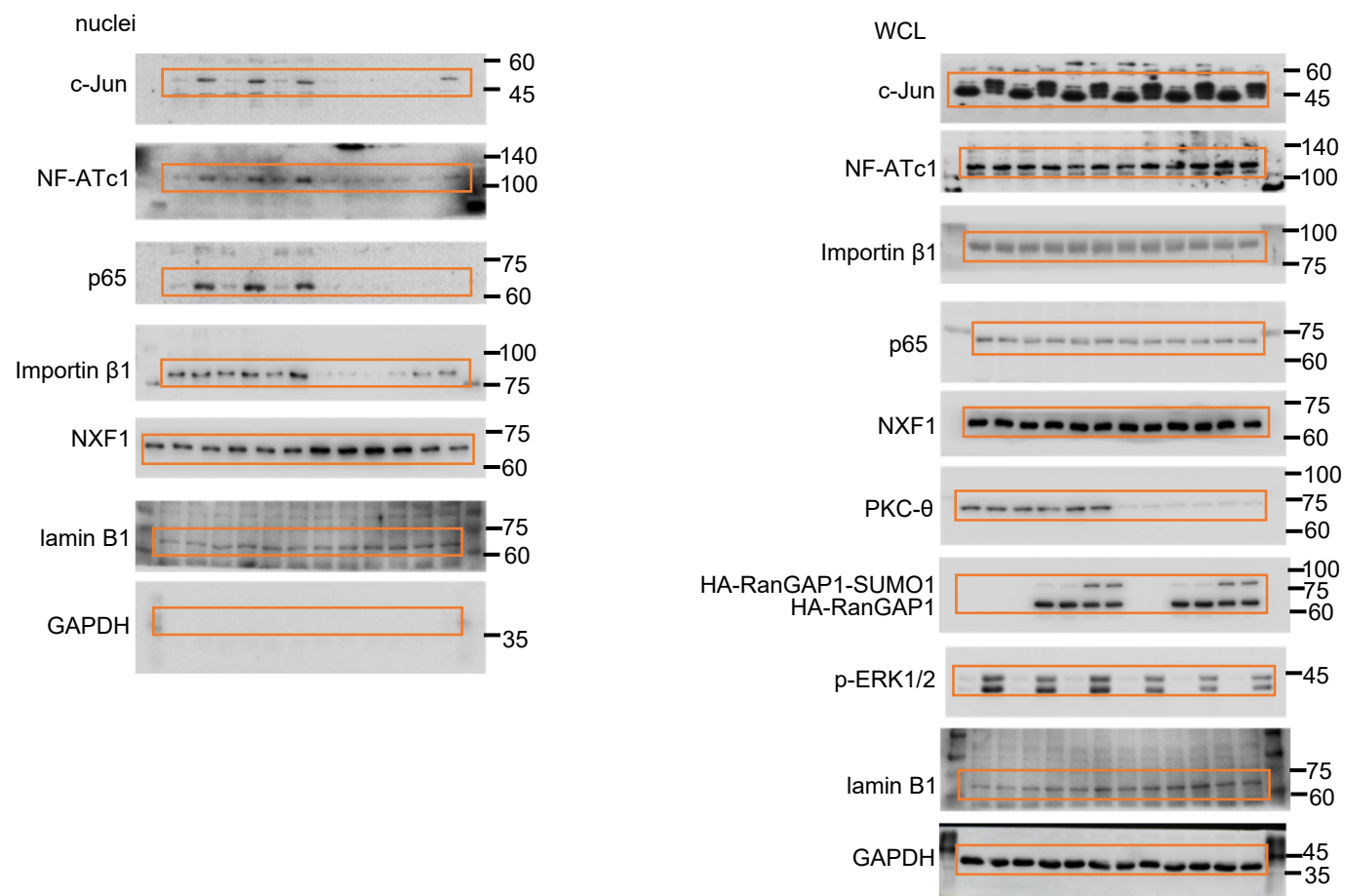

Supplement: Figure 8—source data 1. [file elife-67123-fig8-data1.pdf]
